# Manufacturing Bulk Nanocrystalline Al-3Mg Components Using Cryomilling and Spark Plasma Sintering

**DOI:** 10.3390/nano12203618

**Published:** 2022-10-15

**Authors:** Amanendra K. Kushwaha, Manoranjan Misra, Pradeep L. Menezes

**Affiliations:** 1 Department of Mechanical Engineering, University of Nevada, Reno, NV 89557, USA; 2 Department of Chemical and Materials Engineering, University of Nevada, Reno, NV 89557, USA

**Keywords:** nanocrystalline, aluminum, cryomilling, sintering, synthesis, characterization, mechanical properties

## Abstract

In the current study, pure aluminum (Al) powders were cryomilled with and without 3 wt.% pure magnesium (Mg) dopant for varying durations followed by spark plasma sintering (SPS) of powders to prepare bulk components with superior mechanical properties. The crystallite sizes were determined for powders and the bulk components by analyzing the X-ray diffraction (XRD) spectrum. The calculations indicated a reduction in crystallite size with the increase in the cryomilling duration. The results also showed a more significant decrease in the crystallite sizes for Al-3Mg samples than that of pure Al. The changes in the surface morphology of powders were characterized using scanning electron microscopy (SEM). The elemental mapping analysis at nanoscale was carried out using Energy-dispersive X-ray spectroscopy (EDX) in Scanning transmission electron microscopy (STEM). The mechanical properties of the bulk components were assessed using a Vickers Microhardness tester. The test results demonstrated an improvement in the hardness of Mg-doped components. Higher hardness values were also reported with an increase in the cryomilling duration. This article discusses the mechanisms for the reduction in crystallite size for pure Al and Al-3Mg and its subsequent impact on improving mechanical properties.

## 1. Introduction

Conventional polycrystalline materials have been studied by researchers for structural and industrial applications over the past few decades. However, the advancement in technology and material science in the past few decades and the ever-increasing demand for improved mechanical properties for diverse applications have attracted many scholars to pursue research in this area. Typically, in polycrystalline materials, the deformation mechanism governs the principle mechanical properties, which in turn largely depends on the movement of dislocations through the crystallographic planes. As the grain size reduces below 100 nm, the dislocation movement becomes more prominent [1]. Due to their incredibly small size, a large volume fraction of dislocations gets piled up at the grain boundaries affecting the deformation mechanism, which imparts unique and superior properties to these materials [2]. Therefore, the mechanical properties of polycrystalline materials can be improved by reducing the crystallite size [3]. The correlation between the mechanical properties and crystallite size is given by the Hall-Petch Equation (1):

(1) $$\sigma_{y}={\;\sigma }_{0}+{\;\mathrm{kd}}^{-1/2}$$

This relation suggests that the material’s flow stress (σ_y_) is inversely proportional to the crystallite size (d). Here, σ_0_ is the frictional stress, and k is a constant. It is also crucial to remember that this decrease becomes quite substantial below 100 nm grain size since the flow stress is inversely related to the square root of crystallite size. As a result, the equation becomes extremely sensitive at smaller crystallite size values. It is also crucial to understand that this synergistic activity diminishes beyond 10 nm since amorphous grain boundaries are now a sizable percentage of the material [4].

The concept of nanocrystalline (NC) materials was first introduced by Birringer et al. [5] in 1984. NC materials are defined as single or multiphase solids with average crystallite sizes lower than 100 nm. This NC structure causes a significant volume proportion of dislocations to concentrate close to the grain boundaries, which impedes dislocation motion and creates internal flow stresses. Therefore, this increased flow stress provides superior properties to NC materials compared to conventional polycrystalline materials. Recent research on NC materials has revealed notable advancements in mechanical characteristics, including high tensile strength [6,7,8], high hardness [9,10], outstanding fatigue resistance [11,12], and exceptional wear resistance [13,14]. There are several methods for manufacturing NC materials such as high energy ball milling (HEBM) [15,16,17], cryomilling [18,19,20,21], rapid solidification [22], spark erosion [23], chemical precipitation [24], chemical vapor deposition (CVD) [25], etc. For solid-state powder processing, mechanical attrition methods such as HEBM and cryomilling are very effective in large-scale production [26,27]. However, the powdered metal is highly susceptible to contamination during the milling process. The erosion of the milling media/milling chamber occurring during the repeated impact of the milling media with each other and the walls of the milling chamber may lead to contamination [28]. The metal powders can also undergo rapid atmospheric oxidation to form metal oxides. Studies have shown that the use of milling at cryogenic temperatures can help to minimize atmospheric oxidation as the rate of oxidation is extremely low at these extreme temperatures [28]. As compared to HEBM, milling at cryogenic temperatures also promotes early fracture of particles and requires much lesser milling time (rapid grain refinement) thereby reducing the effective interaction time of milling media with the milled powders. This helps prevents the contamination of the NC powders [29].

Although NC materials possess such a high potential for improving their properties, their usage is currently limited in practical applications. Due to smaller grain sizes, a large volume fraction of high-energy grain boundaries gets accumulated in a small region. Since a coarser grain boundary has less stored energy due to a smaller border region compared to an NC grain structure, the grain boundaries attempt to become thermodynamically stable by undergoing grain coarsening. As a result, NC materials are highly unstable as the grain coarsening can readily occur at much lower temperatures. Kumar et al. [30] developed an environmentally friendly way of producing a large quantity of NC Al that is stable up to 100 °C. They performed cryomilling for 6 h 30 min to obtain crystallite size of 10–15 nm. They also showed that upon annealing at temperatures of 150 °C and above the grain coarsening occurs and the crystallite size increases to greater than 100 nm which is beyond the NC regime. Tang et al. [31] processed Al 5083 composite with SiC_p_ by cryomilling followed by hot isostatic pressing (HIP) to prepare bulk components. They achieved a crystallite size of 30 nm for the powdered samples after cryomilling however the HIP process led to extensive grain coarsening increasing the average crystallite size to around 200 nm. They also reported a crystallite size of about 700 nm in the inter-particle region. Kishimoto et al. [32] also worked with mechanical alloying of ferritic alloys and carried out the HIP process to obtain bulk samples. They reported a tremendous increase in the crystallite size varying from 25 nm to 500 nm. To cater to this, the spark plasma sintering (SPS) process can be employed, which is a modified form of the HIP process [33,34,35]. In this process, the powdered sample in the die is subjected to a uniform uniaxial pressure and the pulsed current concurrently for the densification process. This allows for rapid heating with a uniform heat distribution allowing for a uniform grain structure throughout the sample [35,36]. With uniaxial pressure, densification of the powders can be achieved at much lower temperatures by SPS as compared to the HIP process, thereby allowing for lesser grain coarsening [37]. Due to the pulsed current, an electric discharge is generated which aids in the breakdown of any oxide layer on the sample [38]. Ye et al. [26] cryomilled commercial grade Al 5083 alloy for 8 h and then performed SPS to prepare bulk components. The SPS process was carried out at 350 °C at a load of 908 kg for 60 s. Although they were able to obtain a lower crystallite size of 25 nm for the cryomilled powders, the crystallite size increased drastically to 51 nm after the SPS process. They concluded that the drastic increase in the crystallite size is due to the elevated temperatures during the SPS process. 

To minimize the grain coarsening for NC materials, the excess grain boundary energy needs to be stabilized without compromising the crystallite size of the materials. Recent studies have demonstrated that preferential doping of the high-energy grain boundaries with secondary phase dopants can reduce the interfacial energy at the grain boundaries [39]. It has also been demonstrated that the second phase material at the grain boundaries aids in pinning the grain boundaries, reducing their energy and their susceptibility to undergo grain coarsening [40]. Therefore, the current experimental work validates a hypothesis that the Mg dopant can preferentially segregate at the grain boundaries and can lower the energy of grain boundaries thereby providing thermodynamic stability to the grain boundaries [41]. The Mg as a dopant in limited quantity gets accumulated at the grain boundaries of pure Al and provides an obstruction to the grain growth. This can be achieved by cryomilling pure Al with a limited quantity of pure Mg instead of using an Al-Mg alloy such as Al 5083 alloys.

In the current experimental work, the cryomilling process was utilized to reduce the crystallite size of powders. Mg was added as a dopant in Al powders to stabilize the high-energy grain boundaries during cryomilling to hinder grain growth. The crystallite size and the morphology of the powders and the SPS components were examined using scanning electron microscopy (SEM) and X-ray diffraction (XRD) techniques. The presence of Mg at the grain boundaries of Al was analyzed using Energy-dispersive X-ray spectroscopy (EDX) elemental mapping in Scanning transmission electron microscopy (STEM). The hardness of the bulk SPS sample was determined using Vickers microhardness testing. This article also discusses the inherent mechanism during the cryomilling process and the effect of cryomilling time on the changes in crystallite size. The effect of the Mg dopant on the stability of the high-energy grain boundary in NC Al powders and the SPS components is also examined in this article.

## 2. Materials and Methods

### 2.1. Sample Preparation

**Figure 1:**
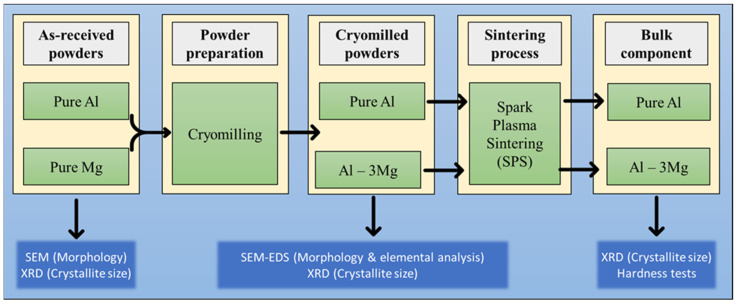
Process flow diagram for the manufacturing of bulk components using cryomilling and SPS.

In the current research, commercial-grade Al and Mg powders from Alfa Aesar were first cryomilled followed by the SPS consolidation process to produce bulk components. The experimental process consists of several material preparation and characterization phases. Figure 1 shows the general process flow diagram for manufacturing bulk components and the characterization methods for each step.

**Figure 2:**
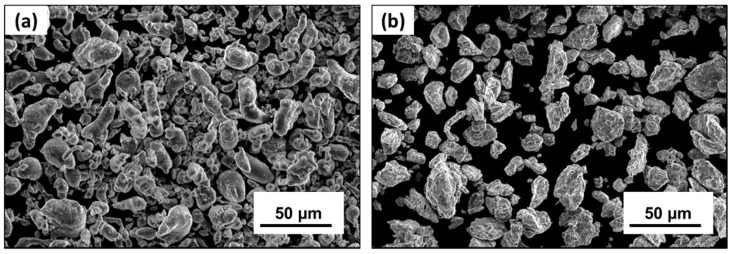
SEM images showing the surface morphology and structure of (**a**) Pure Al and (**b**) Pure Mg powders.

In this experiment, the as-received powders had a purity of 99.5% for pure Al and 99.8% for Mg powders. Both the powders were in the size range of –325 mesh (less than 45 µm). The as-received Al powders particles were irregularly shaped with a cylindrical morphology, as shown in Figure 2a. The surface of the pure Al powders had a smooth surface feature. Figure 2b shows the morphology of the as-received Mg powder particles. Like the as-received Al particles (Figure 2a), Mg powders are irregularly shaped but have a rougher surface texture than pure Al.

For the cryomilling process, the Al powders were mixed with 3 wt.% of Mg powders thoroughly before adding them to the attrition chamber. To prevent powder agglomeration, 0.1 wt.% stearic acid (C_18_H_36_O_2_) was also added to the chamber along with the constituent powders. The cryomilling was performed using a modified Union Process 01-ST attritor mill. The cryomilling was performed using spherical stainless-steel milling media (6.3 mm diameter) at a ball-to-powder ratio of 30:1 with a continuous supply of liquid nitrogen (LN2) at −190 °C. The process parameters for the cryomilling process are listed in Table 1. The milling was performed for 4 h and 6 h to understand the effect of milling duration on morphology and mechanical properties. This is an optimum duration of cryomilling to obtain a maximum reduction in crystallite size with economic energy consumption. Cryomilling beyond 6 h duration does not change the crystallite size considerably due to repeated agglomeration of particles. Therefore, cryomilling beyond 6 h would not be an energy-efficient process [42]. After the milling process, the chamber is transferred to the glove box and the powders are extracted.

**Figure 3:**
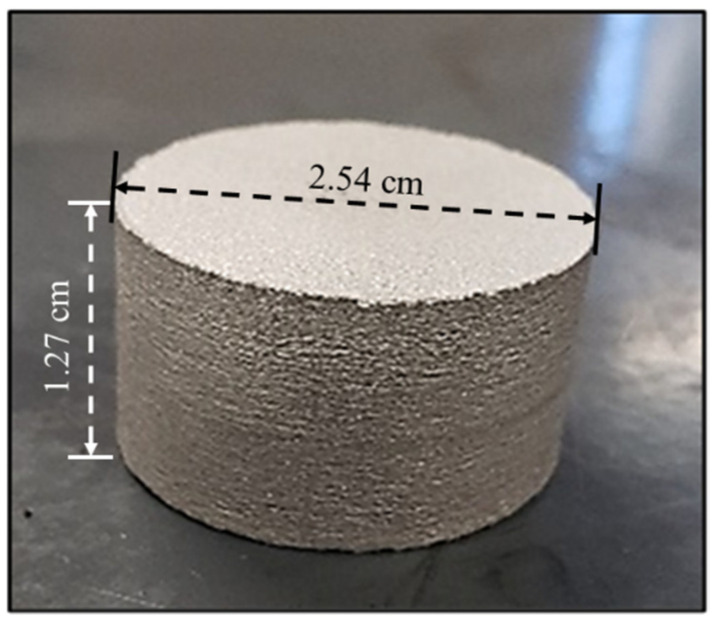
Manufactured Al-3Mg SPS component.

For the consolidation process, the unmilled and the cryomilled powders were compacted using a 2.54 cm mold assembly in a 211XL SPS machine at California Nanotechnologies (Cerritos, CA, USA). The molds were filled with 20 g powder samples and compacted at 500 °C under 100 MPa pressure. Based on preliminary tests, the unmilled and cryomilled samples were sintered for 30 min and 15 min, respectively. Figure 3 shows the prepared SPS component out of the die assembly. The manufactured cylindrical component is 1.27 cm in height and 2.54 cm in diameter. This component is cut, mounted, and polished appropriately to perform characterization studies.

### 2.2. Characterization Methods

The powders and the SPS samples in the current study were characterized using various characterization techniques. To analyze the changes in the morphology of the powders upon cryomilled, both milled and unmilled powders were examined under SEM (FEI Quanta 600 FEG) equipment. Energy dispersive x-ray spectroscopy (EDX) area analysis was also performed on the powder surface to evaluate the presence of Mg in individual particles. The elemental mapping and composition of the powders at the nanoscale were determined using a TEM (JEOL 2800 STEM FE) coupled with dual EDX detectors.

A Bruker D2 Phaser advanced X-ray diffractometer was used to determine the average crystallite size of the samples. To ascertain the composition, the XRD diffraction spectrum from the diffractometer was examined and compared with the reference peak. The distinctive peaks in the XRD spectrum were analyzed to calculate the crystallite size using the Williamson-Hall equation. The Williamson-Hall method states that the total broadening $\left(\beta_{T}\right)$ of the peaks in XRD is the arithmetic sum of the broadening due to strain $\left(\beta_{\varepsilon }\right)$ and broadening due to crystallite size $\left(\beta_{D}\right)$. The total broadening (β_T_) for the Williamson-Hall equation is given by the following Equation (2):

(2) $$\beta_{T}\mathrm{Cos}\theta =\varepsilon \;\left(4\mathrm{Sin}\theta \right)+\frac{K\lambda }{D}$$

Here, K is Scherrer’s constant, λ is the wavelength of the X-ray, D is the crystallite size, ε is the strain, and θ is Bragg’s angle. This equation is similar to the equation of a line y=mx+C. Plotting the curve for the equation for different values of FWHM and θ with $\beta_{T}\mathrm{Cos}\theta$ on the Y-axis and 4Sinθ on the X-axis results in a straight line. The slope of this straight-line fit is the strain (ε), and the y-intercept is $\frac{K\lambda }{D}$. The value for K is 0.94 for Al, and the wavelength λ is 0.154178 nm for the Cu tube in XRD. In this work, the FWHM and 2θ were determined for the first 5 peaks to evaluate the crystallite sizes.

Vickers microhardness tests were conducted on the mounted and polished SPS samples to evaluate the mechanical properties of the components. The tests were conducted on TUKON 1202 Microhardness tester equipment at a load of 0.050 Kg for 10 s loading time. A total of 5 indents were made using a diamond indenter for each sample, and the average microhardness was calculated.

## 3. Results and Discussion

### 3.1. Cryomilled Powders

#### 3.1.1. Effect of Cryomilling Duration

**Figure 4:**
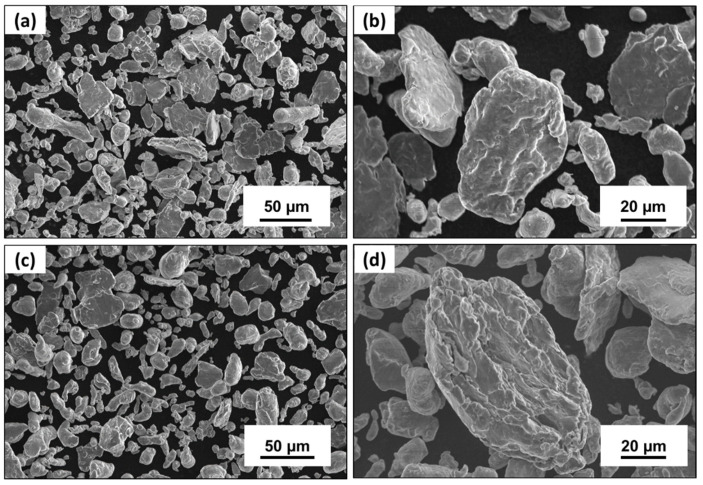
SEM micrographs for Al-3Mg 4 h cryomilled sample showing (**a**) particle morphology and (**b**) shearing and fracture on a single cryomilled particle. The comparative SEM micrographs for 6 h cryomilled sample’s (**c**) morphology and (**d**) fracture surface.

The cryomilling process results in the shearing, fracturing, and cold welding of the pre-mixed Al-3Mg powders in the cryogenic chamber. This results in changing the shape and size of the particles. The morphology of the 4 h cryomilled powders is shown in Figure 4a. It can be confirmed from the SEM image that the cylindrical Al powders with smoother surface textures have now deformed and flattened into irregularly shaped fragments of varying sizes. Figure 4b shows an individual particle for the Al-3Mg cryomilled for 4 h. The particle shows deformation marks formed by repetitive shearing and cold welding of the particles during the cryomilling process. The effect of the cryomilling duration on the particle size and shape was studied by comparing the morphology of powders for different cryomilling durations. Figure 4a shows the morphology of 6 h cryomilled powders. It can be confirmed by comparing the SEM images that after initial deformation and flattening (Figure 4a), the powders tend to start agglomerating into a much rounder shape after 6 h of milling (Figure 4c). Figure 4d shows the enlarged view of 6 h cryomilled individual particles. The surface topography of this particle shows much more striations and cold-welding marks than the 4 h cryomilled particle (Figure 4b).

**Figure 5:**
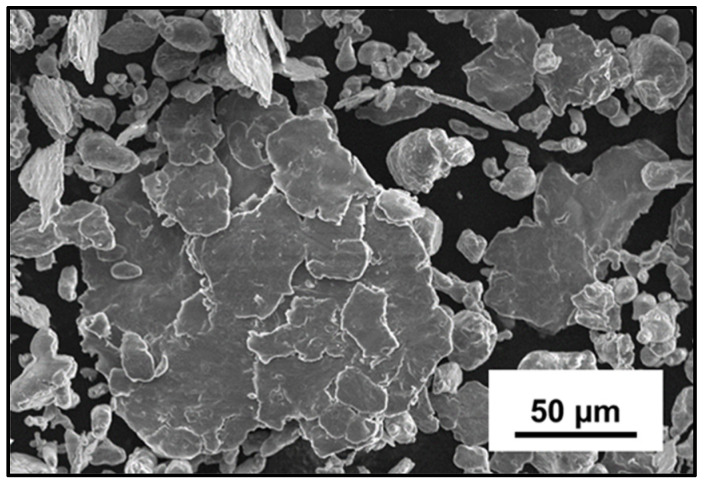
SEM micrograph showing agglomeration of individual sheared and fractured particles to form a larger particle for 4 h cryomilled Al-3Mg powder sample.

During the continuous fracturing and cold welding of the powder particles, some particles undergo massive agglomeration to form a bigger chunk. Despite the addition of the stearic acid, a few particles stick to other particles due to cold welding causing agglomeration. However, these agglomerated particles are again broken down into smaller particles that might be cold welded together to form significantly larger agglomerations. This process continues during the entire duration of the cryomilling process. Figure 5 shows an individual agglomerated particle formed by cold welding of sheared and fractured particles for 4 h cryomilled Al-3Mg powders.

**Figure 6:**
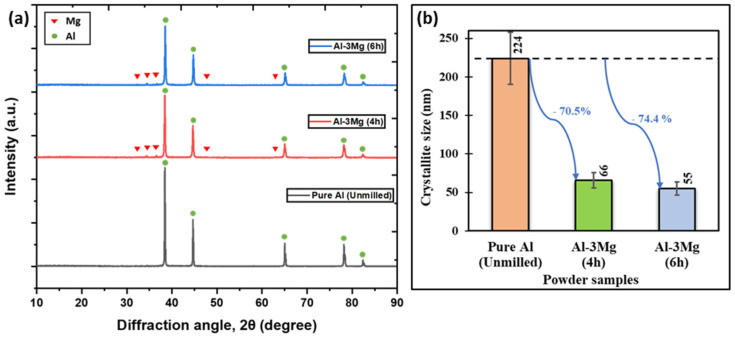
(**a**) XRD spectrum for Pure Al, Al-3Mg 4 h, Al-3Mg 6 h cryomilled powder samples and the (**b**) plot showing the effect of cryomilling duration on the crystallite size of powders.

One of the most essential characteristics of NC materials is crystallite size below 100 nm. However, the goal is always to minimize the crystallite size as much as feasible to get a superior improvement in mechanical characteristics which is governed by the Hall-Petch equation. The NC structure of the as-prepared samples was confirmed by performing the crystallite size analysis on the XRD spectrum. Figure 6a shows the XRD spectra for the unmilled pure Al, Al-3Mg 4 h cryomilled and, Al-3Mg 6 h cryomilled powders. The characteristic XRD peaks for the Mg and Al occurring at specific diffraction angles (2θ) are indicated in the spectrum. The crystallite size of each powder sample was determined using the Williamson-Hall method by evaluating the broadening of characteristic peaks in the XRD spectrum. The crystallite size of powders upon cryomilling decreases with the increase in the cryomilling duration. Figure 6b shows the drastic reduction in the crystallite size with the increase in the cryomilling duration. The crystallite size decreases by 70.5% from 224 nm to 66 nm for 4 h of cryomilling and a decrease of 74.4% to 55 nm for 6 h of cryomilling. Although the decrease in the crystallite size seems low from 4 to 6 h of cryomilling, the decrement is still relevant in improving the mechanical properties. As per the Hall-Petch relationship, the slightest decrease in the crystallite size at lower values can exponentially enhance the mechanical properties.

#### 3.1.2. Effect of Mg Doping

**Figure 7:**
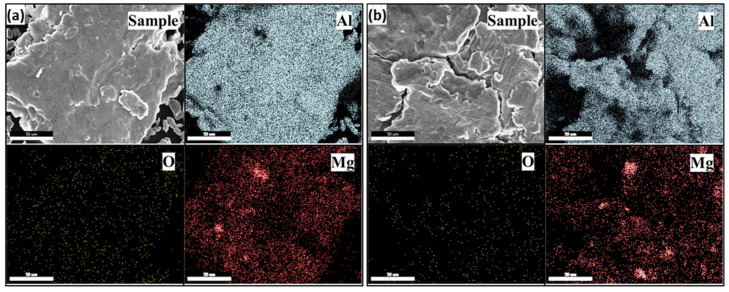
Plot showing SEM-EDX elemental mapping for (**a**) 4 h and (**b**) 6 h cryomilled Al-3Mg powder samples.

The surface and textural characteristics of the cryomilled particles were revealed by particle morphology analysis. The XRD characterization also showed a decrement in the crystallite size with an increase in cryomilling duration. In the current manufacturing approach, a secondary dopant material is added during the cryomilling process. Therefore, it is equally important to analyze the presence of the dopant in the finished cryomilled powders. EDX investigations were conducted on the powder particles for different cryomilling duration to observe the presence of dopant in the cryomilled samples. Figure 7a,b shows the SEM-EDX elemental mapping for 4 h and 6 h cryomilled Al-3Mg powders particles. The EDX mapping shows the distribution of Mg element in the matrix of Al for both samples. The map also indicates the presence of oxygen element in trace amounts which occurs due to the formation of Al and Mg oxides on the particle surface.

**Figure 8:**
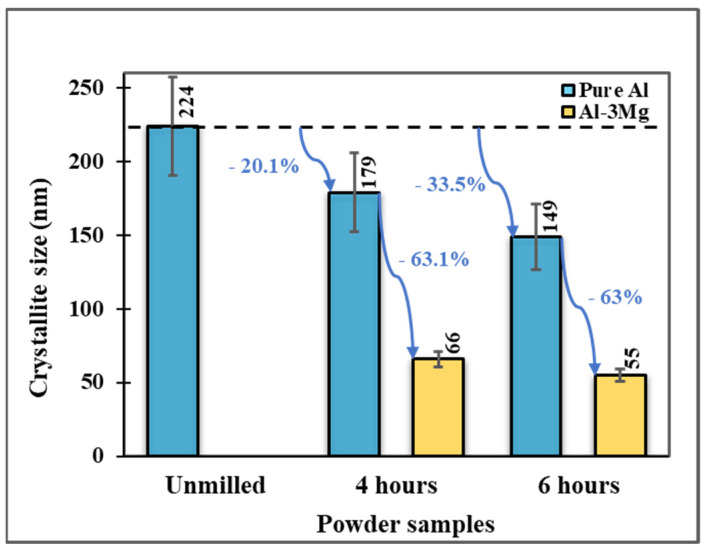
Plot showing the effect of Mg doping on the crystallite sizes for pure Al and Al-3Mg for varying cryomilling times.

To better understand the effect of Mg doping on the crystallite size and subsequently their mechanical properties, cryomilling was performed with and without Mg dopant for Al powders. Figure 8 shows a comparative plot for the crystallite size analysis for pure Al and Al-3Mg cryomilled for different durations. These results can be interpreted in 2 ways (1) reduction in crystallite size with an increase in cryomilling duration, and (2) greater reduction in the crystallite size for Mg-doped Al powders as compared to pure Al. It is already shown that the crystallite size reduces with an increase in cryomilling duration which is confirmed here in pure Al as well. For 4 h of cryomilling, the crystallite size reduces to 66 nm for Al-3Mg compared to 179 nm for pure Al. Similarly, for 6 h of cryomilling, the crystallite size for Al-3Mg reduces to 55 nm compared to around 149 nm for pure Al. This change in the crystallite size is shown to have a much more significant effect on improving the mechanical properties at lower crystallite sizes.

**Figure 9:**
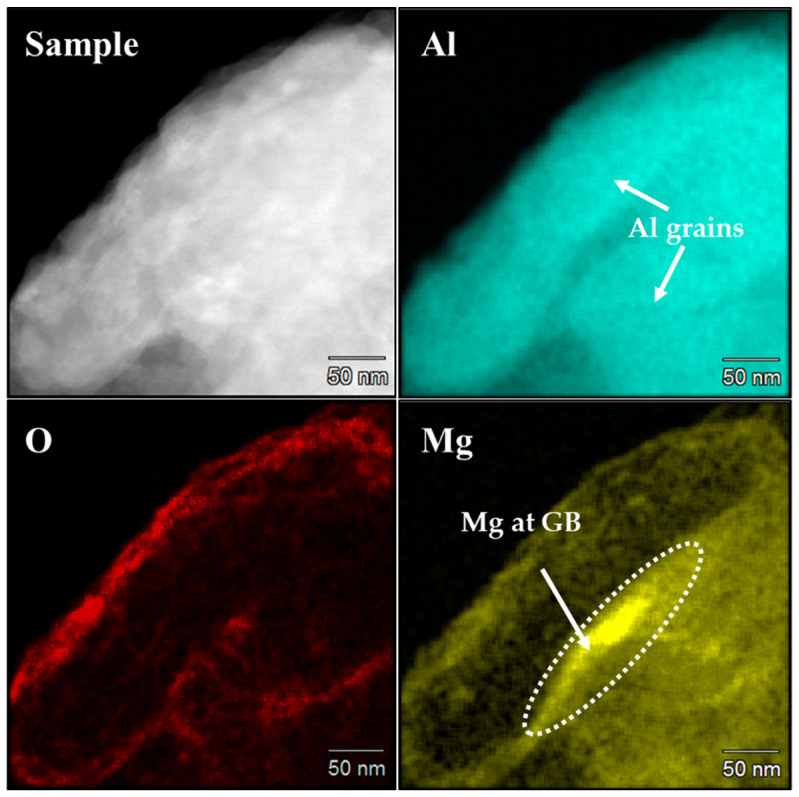
STEM-EDX plot showing the concentration of Mg dopant at the grain boundaries of Al grains.

It is evident that the presence of Mg allows for achieving a much lower crystallite size (Figure 8). The cryomilled Al undergoes a lot of straining with the reduction in crystallite size. As a result, these crystallites have a higher grain boundary energy and are thermodynamically unstable. This implies that at elevated temperatures the grain coarsening would occur much more easily to lower the energy of grain boundaries increasing the crystallite size. Therefore, a secondary dopant is added to the matrix to lower the energy of the grain boundaries. Figure 9 shows the TEM-EDX elemental mapping of the crystallite showing the presence of element Al in each grain. The maximum concentration of Mg can be seen accumulating at the grain boundaries. Adding Mg as a dopant allows for Mg to preferentially segregate out at the grain boundaries of Al to lower the energy of the high-energy grain boundaries. Studies have shown that a certain low solute content (%) at the grain boundaries aided in lowering the Gibbs free energy at the grain boundaries. Furthermore, for positive enthalpy of mixing (kJ/mol) to determine the stability of the Al-Mg system, the enthalpy of segregation (kJ/mol) is higher for Al-Mg binary system to form a stable NC structure [40,41]. Therefore, the Mg has a higher tendency to segregate out at the grain boundaries and help in lowering the energy at the grain boundaries, thereby reducing their susceptibility to undergo grain coarsening.

### 3.2. Spark Plasma Sintered Samples

#### 3.2.1. Effect of SPS on Crystallite Size of Sintered Components

The SPS process was carried out on the unmilled & cryomilled powder samples to obtain bulk components. In this process, the powders were subjected to heat and pressure in a die assembly to produce cylindrical consolidated samples, 2.54 cm in diameter and approximately 1.27 cm in height. The density of the SPS samples was determined using the Archimedes principle. The measured density (ρ_ex_) was determined as 2.6757 g/cm^3^, 2.6260 g/cm^3,^ and 2.6199 g/cm^3^ for unmilled pure Al, Al-3Mg 4 h cryomilled, and Al-3Mg 6 h cryomilled SPS samples. All the produced parts had a density equal to ~99% of the theoretical density. The theoretical density (ρ_th_) of the Al-3Mg was determined as 2.6509 g/cm^3^ using the rule of mixtures for pure Al (2.699 g/cm^3^) and pure Mg (1.738 g/cm^3^). These results indicated that the manufactured SPS samples were homogenous with little or no porosity.

**Figure 10:**
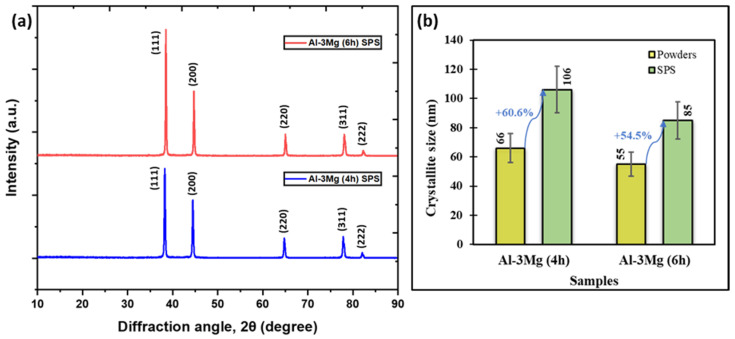
(**a**) XRD spectrum for Al-3Mg 4 h, Al-3Mg 6 h SPS samples and the (**b**) plot showing the effect of the SPS process on the crystallite sizes for pure Al and Al-3Mg samples for varying cryomilling duration.

To understand the effect of the SPS process on the crystallite size of sintered components, each SPS sample was analyzed using XRD. Figure 10a shows the XRD spectra for Al-3Mg 4 h cryomilled and 6 h cryomilled SPS samples. The figure also shows the characteristic peaks for different crystallographic planes for pure Al. These crystallographic planes were determined by comparing the obtained XRD peaks with the peaks in the JCPDS database for pure Al. The FWHM determined at each crystallographic plane’s XRD peak is used to calculate the crystallite size using the Williamson-Hall equation. Figure 10b shows the crystallite size measurements for the powders and SPS’ed samples for different milling duration. It is clear from the plot that the crystallite size increases during the SPS process due to the increase in temperature leading to grain coarsening. The crystallite size for Al-3Mg 4 h cryomilled sample showed an increase of 60.6% upon SPS from 66 nm to 106 nm compared to the powder samples. The Al-3Mg 6 h cryomilled samples showed an increment of 54.5% from 55 nm to 85 nm. Although there is an increase in the crystallite size, the manufactured SPS samples have a stable crystallite structure and a low crystallite size. Apart from lowering the high energy of the grain boundaries, the presence of Mg dopant at the grain boundaries also acts as a pinning site which limits the coarsening of the grain structures at elevated temperatures during the SPS process (Zener pinning). Therefore, the obtained SPS components have a NC structure which would help provide superior mechanical properties to the manufactured parts. 

#### 3.2.2. Mechanical Properties

**Figure 11:**
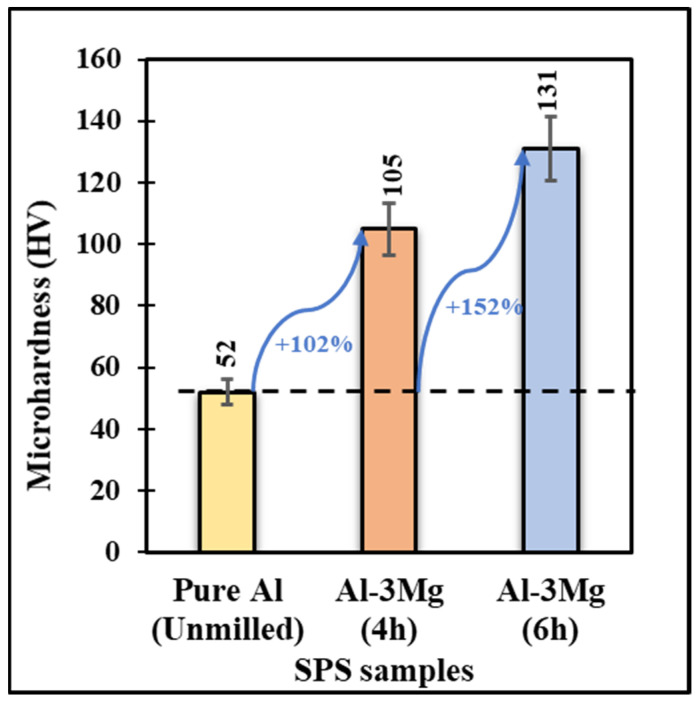
Vickers microhardness comparison for Al-3% Mg samples cryomilled for 4 h and 6 h with the pure Al SPS samples.

It is evident from the XRD analysis that there is a more significant reduction in crystallite size for Al-3Mg samples cryomilled for a longer duration. It is well known that the Hall-Petch relationship correlates the change in crystallite size with the mechanical properties. Therefore, to understand this correlation, Vickers microhardness tests were conducted on the bulk consolidated samples for varying cryomilling durations. Figure 11 shows the variation of Vickers microhardness of Al-3Mg samples cryomilled for different durations compared to unmilled pure Al. The microhardness for unmilled pure SPS Al is 52 HV. For 4 h of cryomilling, the microhardness of Al-3Mg is determined at 105 HV, which is a 102% increase as compared to the pure Al sample. For 6 h cryomilled samples, the hardness is determined at 131 HV, which is almost 152% greater than that of the pure Al. This drastic increase in the microhardness correlates directly with the decrement in the crystallite size at lower values in the NC regime. Compared to the Al-3Mg 6 h cryomilled sample, which has a Vickers microhardness value of 131 HV, a standard Al 5083 alloy has a Vickers microhardness value of 70 HV. Several studies have been conducted on Al alloys, such as Al 5083, to improve their properties. Baig et al. [43] processed Al 5083 alloy by equal-channel angular pressing (ECAP), in which the microstructure is altered using the severe plastic deformation (SPD) method. They achieved a microhardness of 75 HV for the 1st pass to 91 HV in the 3rd pass for the Al 5083 alloy. The decrement in crystallite size can accommodate more grains in a much smaller area. Therefore, the grain boundaries of such closely packed grain structures serve as a barrier to the migration of dislocations inside the grain structure of the NC materials. This significant accumulation of dislocations decreases the material’s plastic flow, hence enhancing the material’s strength and microhardness. As a result, NC Al-3Mg shows superior mechanical properties for lower crystallite sizes [44].

## 4. Conclusions

The current experimental work demonstrated the manufacturing of bulk NC Al-3Mg components using cryomilling and SPS processes using a novel technique. Unlike the preceding research, where Al-Mg alloys were cryomilled to reduce the crystallite size, the current work involves cryomilling a mixture of Al and Mg without alloying them. As hypothesized, this allowed the Mg to segregate at the grain boundaries of pure Al, lowering the grain boundary energy and forming a stable system. The manufactured components exhibited superior mechanical properties compared to pure Al due to a greater reduction in the crystallite size in the NC regime. The Al-3Mg components were also able to reduce the effect of coarsening. Therefore, this study provides a good insight into the effect of Mg in reducing the crystallite size during the cryomilling process, which can be used to produce bulk components with superior mechanical properties. This insight may also be used to prepare NC bulk components with other alloys with many superior properties. The current investigation leads to the following conclusions:

1. The reduction in the crystallite sizes for Al-3Mg can be attributed to (1) an increase in cryomilling duration and (2) the addition of Mg dopant.
2. Al-3Mg powders showed a 74.4% decrement in crystallite size after 6 h of cryomilling which is greater than that achieved with pure Al. This decrease is attributed to the preferential segregation of Mg dopant at the grain boundaries of pure Al, stabilizing its grain boundary energy and preventing grain growth.
3. The crystallite size increases during the SPS process due to the elevated temperatures leading to grain coarsening. The Al-3Mg samples showed a lower increase in the crystallite size after SPS indicating that the Mg helped in limiting the effect of grain coarsening by pinning the grain boundaries.
4. The Al-3Mg SPS samples showed a 152% increase in microhardness compared to the unmilled pure Al SPS samples. This increase in property can be attributed to the lower crystallite size of the SPS’ed samples.

## Figures and Tables

**Table 1:** Process parameters for the cryomilling process.

Process Parameters	Values
Speed	180 rpm
Ball to powder Ratio	30:1
Milling Media diameter	6.3 mm
Milling Time	4 and 6 h
Milling Temperature	−190 °C

## Data Availability

Not applicable.
